# The prevalence of retinal lesions in septic ICU patients - a cross-sectional, observational study

**DOI:** 10.1186/2197-425X-3-S1-A875

**Published:** 2015-10-01

**Authors:** G Chanchalani, P Jiandani, AS Ansari, S Mehta

**Affiliations:** Action Cancer Hospital, Department of Critical Care, Delhi, India; Wockhardt Hospital, Mumbai, India; Lilavati Hospital and Research Centre, Mumbai, India

## Introduction

Invasive bacterial infections are known to involve the retina, but the utility of ophthalmologic consultation in the critical care setting as a diagnostic tool are not well understood.

## Objectives

To understand better the frequency of retinal lesions, their diagnostic significance, in patients with bacteremia, and the utility of fundus examination in the critical care setting.

## Methods

We performed funduscopic examinations on 73 patients with clinical history suggestive of infection, on the day of admission to ICU, and reviewed the literature on the association of retinal lesions with disseminated bacterial infection and compared our results with previous prospective reports in the literature.

Enrollment of patients was performed at the ICU of our center over one month. Patients were excluded if they were found to have a non-infectious disease. Patients were excluded if they refused entry or if a history of an eye condition precluded ocular examination. For all patients, a detailed history with emphasis on underlying systemic diseases was taken.

After enrollment, a detailed examination of the posterior pole was carried out by means of indirect ophthalmoscopy by a retinal specialist, who was blinded about the systemic diseases of the patient and the status of bacteremia.

## Results

We found that 25/73 (34%) of the ICU patients had retinal lesions consistent with disseminated bacterial infection. Of these 25, 12 (48%) had clearly sepsis-related retinal lesions, while 13 had 1 or more systemic disease that could have explained their retinal findings (11 diabetic retinopathy; 18 hypertensive retinopathy; 1 Leukemia).

Among the 13 patients with retinal lesions and systemic diseases, 7 had episodes of bacteremia. Nevertheless, it is also possible that at least some of these lesions were due to an intercurrent episode of disseminated bacterial infection.

However, only 14/25(56%) of the patients with retinal lesions had a temporally related episode of bacteremia. Bacteremia was also noted in 22 patients with normal fundoscopic appearance.

Only 14 patients out of 36 (38%) with bacteremia had positive retinal findings consistent with disseminated bacterial infection. Whereas, similar retinal lesions were observed in 11 out of 37 patients (29%) who were septic but non-bacteremic.

## Conclusion

We conclude, retinal lesions are quite common in the critically ill patient. While lesions such as cotton wool spots, hemorrhages, and Roth spots may represent infection, the data suggest that these lesions are more often related to an underlying systemic illness. Taken together, these data suggest that ophthalmologic consultation in the ICU setting has little value for the diagnosis of occult sepsis.

**Table 1:** Table 1

Intra retinal hemorrhages	13
Venous congestion	5
Cotton wool spots	5
Roth spots	4
Choroiditis	2
Disc hemorrhages	1
Disc edema	1
Putchner's retinopathy	1

*[list of ophthalmoscopic findings observed]*
